# Screening of Wilson’s disease in a psychiatric population: difficulties and pitfalls. A preliminary study

**DOI:** 10.1186/s12991-017-0142-6

**Published:** 2017-04-04

**Authors:** Caroline Demily, François Parant, David Cheillan, Emmanuel Broussolle, Alice Pavec, Olivier Guillaud, Lioara Restier, MOPSY Consortium, Philippe Bernard, Fabienne Bourdoncle, Patrick Briant, Alain Fouilhoux, Sandrine Foullu, Marie-Hélène Girard-Madoux, Caroline Jeanpierre, Bernard Joli, Marianne Lemarié, Sophie Lonjaret, Jacques Marescaux, Philippe Paulino, Lionel Reinheimer, Jean-Maurice Tarissan, Alain Lachaux, Muriel Bost

**Affiliations:** 1 GénoPsy, Center for the Detection and Management of Psychiatric Disorders of Genetic Origin, Pôle Ouest, Hôpital le Vinatier & UMR 5229 (CNRS & Lyon University), 95 Bld Pinel, 69677 Bron cedex, France; 2 Laboratory of Inherited Metabolic Diseases, Centre de Biologie Est, Hospices Civils de Lyon, Bron, France; 3 National Reference Center for Wilson’s disease, Hôpital Femme Mère Enfant, Hospices Civils de Lyon, Bron, France; 4 Neurology Unit C, Cognitive Neurosciences Center, Hôpital Neurologique Pierre Wertheimer, Hospices Civils de Lyon; Claude Bernard-Lyon 1 University; CNRS UMR 5229, Bron, France; 5 Hôpital Saint Jean de Dieu, Lyon, France; 6 Hepato-Gastroenterology Department, Hôpital Edouard Herriot, Hospices Civils de Lyon, Lyon, France; 7 Gastroenterology, Hepatology and Pediatric Nutrition Department, Hôpital Femme Mère Enfant, Hospices Civils de Lyon, Bron, France; 8 Pharmaco-Toxicology, Biochemistry and Molecular Biology Unit, Hôpital Édouard Herriot, Hospices Civils de Lyon, Lyon, France

**Keywords:** Serum copper, Ceruloplasmin, *ATP7B gene*, Wilson’s disease, Psychiatric disorders, Inborn errors of metabolism, Treatable hereditary metabolic disorders, Copper homeostasis, Copper chelators, Schizophrenia, Bipolar disorders, Alcohol abuse, Mental health, Mental diseases, Etiopathogenesis

## Abstract

**Background:**

Wilson’s disease (WD) is a rare autosomal-recessive, inherited disorder caused by a mutation in the copper-transporting gene *ATP7B* affecting the liver and nervous system. About 30% of patients with WD may initially present with psychiatric symptoms, and diagnosis can be difficult to establish. The objectives of the present preliminary study were [1] to evaluate the relevance of serum copper (Cu) and ceruloplasmin (Cp) measures in hospitalized patients with psychiatric disorders; and [2] to identify possible mutations in the *ATP7B* gene in patients with abnormal biological copper profile.

**Methods:**

All psychiatric patients who participated in this study were hospitalized in Saint-Jean de Dieu Hospital (Lyon, France). Cp was measured by immunoturbidimetry and serum Cu by inductively coupled plasma-optical emission spectrometry. When Cp and serum Cu levels were inferior to, respectively, 0.18 g/L and 0.88 mg/L in combination with atypical psychiatric presentations, complete clinical examinations were performed by multidisciplinary physicians specialized in WD. In addition, mutation detection in the *ATP7B* gene was performed.

**Results:**

A total of 269 patients completed the study. (1) 51 cases (19%) showed both decreased Cp and Cu concentrations. (2) Molecular genetic tests were performed in 29 patients, and one *ATP7B* mutation (heterozygous state) was found in four patients. We identified three different missense mutations: p.His1069Gln, c.3207C>A (exon 14), p.Pro1379Ser, c.4135C>T (exon 21) and p.Thr1434Met, c.4301C>T (exon 21). No pathogenic mutation on either *ATP7B* allele was detected.

**Conclusion:**

Results of Cp and/or serum Cu concentrations below the normal limits are common in patients with psychiatric disorders and nonrelevant and/or informative for the WD diagnosis. WD diagnosis is based on a combination of clinical and biological arguments. Psychiatric patients with suspicion of WD should be evaluated in a reference center.

*Trial registration* CPP Lyon Sud-Est IVNo 10/044, CNIL No DR-2011-470, Afssaps No B100832-40 and CCTIRS No 10.612 bis, registered 8 June 2010

## Background

Wilson’s disease (WD, MIM#27790) is an inherited, autosomal-recessive disorder of copper metabolism. It is caused by mutations in the *ATP7B* gene (MIM#606882), which is located on chromosome 13 [1] and encodes for a membrane copper-transporting ATPase, ATP7B (NM 000053.3). The protein is located on the Golgi membrane and is involved in copper transport across cell membranes. The prevalence of WD is estimated as one in 30,000 in most populations with a carrier frequency of one in 90 [2], but a recent study of abnormal gene frequency points to a possible higher prevalence of 1/7026 [3]. The disease is fatal unless treated by effective medication such as chelators and zinc salts [4]. The initial clinical manifestations are mainly hepatic (40% of cases), neurological (35%), and psychiatric (10%). Hematologic, renal, or ocular (15%) manifestations may also be associated [5, 6]. According to case series, up to one-third of the WD patients may initially present with behavioral or psychiatric abnormalities [7].

When psychiatric manifestations are isolated, WD diagnosis is difficult to establish. Brain magnetic resonance imaging may be normal [8], and Kayser–Fleischer ring may be absent in many psychiatric presentations [9]. WD diagnosis is often delayed when psychiatric symptoms preceded neurological or hepatic involvement [10]. About 20% of patients commonly undergo psychiatric treatment before specific chelation therapy [11, 12]. A wide set of psychiatric, psychological, and psychosocial impairments have been reported. These include intellectual deficiency, confusion, cognitive impairment, dementia, poor scholar performance, anxiety, depression, emotional lability, mania, behavioral abnormalities and personality disorders, schizophrenia-like states, and suicide. A retrospective analysis of patients with various hereditary metabolic disorders including WD showed that psychiatric signs may remain isolated for years before other more specific organic signs appear [13].

In WD patients, an early and accurate diagnosis is the key to effective disease management (refer patients to a reference center; initiate chelator therapy in order to prevent irreversible complications; and provide genetic counseling to the patients and their family). However, WD diagnosis still remains a challenge [14] because (a) care team does not think about this pathology, particularly in psychiatric unit; (b) diagnosis algorithms for WD are based on Leipzig score [15] using a broad combination of biochemical tests (serum ceruloplasmin, 24-h urinary copper excretion, serum-free copper, and hepatic copper), but most of them are difficult to apply to psychiatric patients.

Typically, the biological detection of WD is often based on ceruloplasmin (Cp) and serum copper (serum Cu) measurements (levels of Cp and serum Cu are usually abnormally low). If the results from these tests are abnormal or unclear, then they may be followed by a 24-h urinary copper test to measure copper elimination. In psychiatric patients, this procedure may be associated with practical difficulties, often with incomplete urinary sampling or times errors. The urinary copper/creatinine (Cu/Cr) ratio may be used in replacement.

The main objective of the present preliminary study was to assess the Cp, serum Cu, and urinary Cu/Cr ratio determination in hospitalized patients with psychiatric disorders, in terms of feasibility and relevance. The second objective was to identify possible mutations in the *ATP7B* gene in patients with abnormal biological copper profile.

## Methods

### Patients: psychiatric diagnosis interview, tools, and psychiatric assessment

The study protocol was approved by the competent French data-protection authority (CPP Lyon Sud-Est IVNo 10/044, CNIL No DR-2011-470, Afssaps No B100832-40 and CCTIRS No 10.612 bis), in compliance with French legislation. Written and informed consent was obtained from patients, and from parents or guardians of minors.

Patient inclusion in the study lasted 15 months. A total of three hundred and five patients (16–65 years old) hospitalized for various mental health disorders in Saint-Jean de Dieu Hospital (Lyon, France) were included. The recruitment included all patients (without specific clinical inclusion criteria) already hospitalized and newly admitted patients. Exclusion criteria were emergency conditions, inflammatory or tumoral diseases, and lack of consent. Pregnant women could be enrolled, but data were considered separately. The size of the population in this proof-of-concept study was not designed to provide answers regarding the prevalence of WD among hospitalized patients with psychiatric disorders (which is thought to be higher than that in the general population) [10] but was determined according to the challenge of performing laboratory tests with complete clinical and possible neurological and hepatic assessment in a large sample of hospitalized patients with psychiatric disorders; and the documented variabilities of Cp and serum Cu levels in the general hospitalized population [16, 17].

Two evaluators established consensus diagnoses according to ICD-10 criteria. The diagnosis was systematically assessed by two trained psychiatrists. They used all the information available from direct interviews of patients and examination of case notes. Symptoms were evaluated with M.I.N.I. semi-structured interviews [18]. Information about alcohol or substance abuse, psychiatric morbidity, and treatments was recorded. The patients who reported that they did not use substances were considered as nonusers. A clinical assessment was performed in order to disclose neurological or hepatic signs. Clinical and biological data were compiled into case vignettes, and diagnoses were made blind regardless of biological data.

### Study design

The study is a descriptive and transversal study focusing on the serum Cp and Cu measures in a cohort of hospitalized patients with psychiatric disorders.

According to the literature in general population, Cp and Cu concentration values were classified into three categories [19, 20]:

- above 0.18 g/L (Cp) and 0.88 mg/L (serum Cu): normal values;
- between [0.10–0.18] g/L (Cp) and] 0.50–0.88] mg/L (serum Cu): borderline values;
- lower or equal to 0.10 g/L (Cp) and 0.50 mg/L (serum Cu): critical values.

When Cp and serum Cu levels were inferior, respectively, to 0.18 g/L and 0.88 mg/L in combination with atypical psychiatric and/or hepatic presentations, complete clinical examinations were performed by a psychiatrist, a neurologist, and an hepatologist specialized in WD (National Reference Center for Wilson’s disease, *Hospices Civils de Lyon*, France). Slit-lamp evaluation of the cornea for Kayser–Fleischer rings (copper deposits on the outer rim of the cornea) was performed by an experienced ophthalmologist. Urinary copper-to-creatinine ratio was also used for assessing the copper elimination. In addition, mutation detection in the *ATP7B* gene was performed in a second blood sample.

### Ceruloplasmin and serum copper measurements and urinary copper-to-creatinine ratio

Serum Cp was determined using Pentra 400 analyzer (Horiba) using an immunoturbidimetric assay (DAKO Polyclonal Rabbit Anti-Human Ceruloplasmin). The assay was calibrated using the Protein 1 Calibrator (Siemens) traceable to the ERM^®^-DA470 (CRM 470) [21]. Between-runs imprecision ranged from 4.8% CV (coefficient of variation) at 0.08 g/L to 2.0% CV at 0.33 g/L. The method accuracy was determined using ProBioQual Scheme samples (http://www.probioqual.com).

Serum and urinary Cu concentrations were measured using an inductively coupled plasma-optical emission spectrometer (Vistapro, Varian). Between-runs imprecision ranged from 9.7% CV at 1 mg/L to 8.7% CV at 2.99 mg/L. Urinary copper-to-creatinine ratios were determined in random urinal samples. External quality control was ensured through participation in the CTQ Proficiency Testing Scheme (https://www.inspq.qc.ca/ctq/paqe/pci/description).

### Mutation detection in the ATP7B gene: sequencing and MLPA analysis

Genomic DNA was extracted from 10 mL of EDTA anticoagulated blood using salt precipitation method (Nucleon BACC3 RPN 8512, GE Healthcare).

For mutation analysis, PCR amplification of the 21 exons and flanking regions of the *ATP7B* gene was performed using intronic primer pairs and the PCR conditions previously described [22]. Bi-directional sequencing was performed on AB 3730 Genetic Analyser with dye-termination chemistry, using SeqScape software. Nucleotide changes were detected by comparing the sequence obtained in the chromatogram with the normal gene sequence (NM_000053.3; *Homo sapiens* chromosome 13 complete sequence).

Patients with one mutation in the *ATP7B* gene were tested using MLPA assay (SALSA MLPA KIT Wilson disease, MRC-Holland).

Our laboratory participates in the EMQN, an European interlaboratory quality-control program for mutation detection in WD.

### Statistical analysis

Statistical analysis was performed with MedCalc (version 12.3.0; MedCalc Software, Mariakerke, Belgium). Two-sample group comparisons of median values were assessed with Mann–Whitney test. Multiple-group comparisons were assessed with Kruskal–Wallis test. A value of *p* < 0.05 was considered as statistically significant.

## Results

### Demographic and clinical data

From the population of 305 patients initially included, a total of 269 patients completed the study (mean age of 41 ± 22 years, 118 females and 151 males). Thirty-six patients were excluded from the study because of missing data.

The following ICD-10 diagnoses were observed: schizophrenia (*n* = 116), bipolar disorders (*n* = 37), alcohol abuse (*n* = 37), depressive and/or anxiety disorders (*n* = 27), behavioral or personality disorders (*n* = 27), intellectual disability (*n* = 7), autism (*n* = 5), anorexia nervosa (*n* = 2), cannabis addiction (*n* = 4), and miscellaneous neurological disorders (*n* = 7).

All patients received psychotropic treatment (monotherapy or combination therapy):

- antipsychotics (*n* = 210): olanzapine (*n* = 63), risperidone (*n* = 33), aripiprazole (*n* = 23), cyamemazine (*n* = 27), haloperidol (*n* = 16), clozapine (*n* = 17), loxapine (*n* = 15), and other antipsychotic drugs (*n* = 16).
- anticonvulsants (*n* = 49): valproate (*n* = 32), gabapentin (*n* = 9), clonazepam (*n* = 6), and carbamazepine (*n* = 2);
- antidepressants (*n* = 46): escitalopram (*n* = 25), paroxetine (*n* = 8), venlafaxine (*n* = 6), agomelatine (*n* = 6), and fluoxetine (*n* = 1);
- lithium (*n* = 14).

Other non-psychotropic temporary treatments were not mentioned.

### Serum ceruloplasmin and copper determination

Serum Cp and Cu levels were measured in all the 269 patients.

Median serum concentration was 0.21 g/L (interquartile range [IQR] 0.18–0.25) for Cp and 1.02 mg/L (IQR 0.88–1.17) for Cu.

### Intergroup comparisons

#### Gender

Serum Cp concentrations were significantly lower in male (median 0.20, IQR 0.16–0.23 g/L) than those in nonpregnant female patients (median 0.23, IQR 0.20–0.26 g/L). Similarly, Cu concentrations were significantly lower in male (median 0.93, IQR 0.80–1.12 mg/L) than in female patients (median 1.08, IQR 0.96–1.27 mg/L) (all *p* < 0.0001). In a pregnant woman, serum Cp concentration was 0.42 g/L and Cu concentration was 1.89 mg/L. These values are above the 95th percentiles determined in nonpregnant women (0.37 g/L and 1.85 mg/L for, respectively, Cp and Cu).

#### Psychiatric diagnosis and clinical data

In patients diagnosed with alcohol abuse (*n* = 37), serum Cp (median 0.23, IQR 0.21–0.26 g/L) and Cu levels (median 1.15, IQR 0.97–1.31 mg/L) were slightly increased compared with patients with other diagnoses (Cp: median 0.21, IQR 0.20–0.22 g/L; and Cu: median 1.01, IQR 0.87–1.15 mg/L) (*p* < 0.03, Mann–Whitney test).

**Fig. 1:**
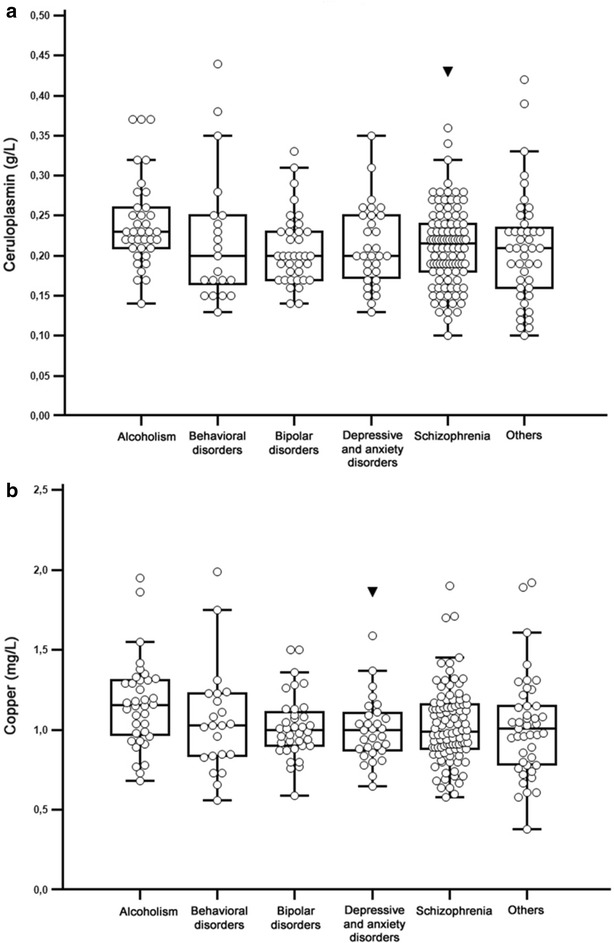
Box plot graphs depicting serum Cp (**a**) and Cu concentrations (**b**) according to psychiatric diagnosis. The median serum Cp and Cu concentrations were not statistically different, except for alcoholism (*p* < 0.05 alcoholism vs. nonalcoholism)

No association between serum Cp or Cu concentrations and psychiatric diagnosis was found in patients diagnosed with schizophrenia, bipolar disorders, depression and/or anxiety, behavioral or personality disorders, and other nonalcohol-related diseases (*p* > 0.9, Kruskal–Wallis test) (Fig. 1a, b). Moreover, no association between serum Cp or Cu concentrations was found in patients with extrapyramidal symptoms (*n* = 5) (*p* > 0.9, Mann–Whitney test). The concentrations found in patient with intellectual deficiency (*n* = 1) and in patient with kidney failure (*n* = 1) were above normal values.

#### Treatment

No association between serum Cp or Cu concentrations and treatment was found in patients under treatment with antipsychotics, antidepressants, and anticonvulsants (*p* > 0.9, Kruskal–Wallis test).

#### Critical and borderline values

About 25% of patients (*n* = 67/269) showed serum Cp concentration <0.18 g/L; 24% (*n* = 66/269) showed Cu concentration <0.88 mg/L; and 19% (*n* = 51/269) showed both decreased Cp and Cu concentrations. Two patients (with psychiatric diagnosis of schizophrenia and intellectual disability) showed critical serum Cp concentrations (0.10 g/L); one of them also showed critical Cu concentration (0.38 mg/L). In these two patients, the urinary Cu/Cr ratio were not increased (6.3 and 1.8 µg/g, respectively, with a median value in a psychiatric population of 7.4 µg/g).

### ATP7B mutation detection

Fifty-one patients (15%) showed both atypical psychiatric signs and Cp ≤ 0.18 g/L (borderline or critical values) and Cu concentration ≤0.88 mg/L. None of them presented with the clinical signs of neurological WD. Among the 29 patients who gave their informed consent for genetic study, four presented with only one *ATP7B* mutation (heterozygous state) identified by direct sequencing and MLPA analysis. Three different missense mutations were identified: p.His1069Gln, c.3207C>A (exon 14); p.Pro1379Ser, c.4135C>T (exon 21); and p.Thr1434Met, c.4301C>T (exon 21), (Table 1). These variants were classified as disease-causing mutations in the HUGO Wilson’s disease database (http://www.uofa-medical-genetics.org/wilson/index.php) and the Alamut or Polyphen sites.

**Table 1:** Clinical and genetic study of psychiatric patients showing Cp < 0.18 g/L and serum Cu levels < 0.88 mg/L

Diagnosis	Associated symptoms	Cp^a^ (g/L)	Copper^b^ (mg/L)	Mutations in *ATP7B* gene
Schizophrenia (*n* = 13)	Extrapyramidal syndrome (*n* = 3)	0.10–0.17	0.60–0.88	*n* = 2 heterozygous mutations p.His1069Gln c.3207C>A (ex14) (Tanzi 1993) and p.Thr1434Met c.4301C>T (ex 21) [34]
Bipolar disorders (*n* = 4)	Extrapyramidal syndrome (*n* = 1)	0.15–0.17	0.72–0.88	–
ASD (n = 3)	Extrapyramidal syndrome (*n* = 1) Kidney failure (*n* = 1)	0.14–0.18	0.65–0.86	–
Personality disorders (*n* = 3)		0.13–0.15	0.65–0.84	n = 1 p.Pro1379Ser c.4135C>T (ex 21) [22]
Neurological disorder (*n* = 2)	Cerebral anoxia (MRI abnormalities in basal ganglia) (*n* = 1)	0.12–0.16	0.60–0.81	*n* = 1 p.Thr1434Met c.4301C>T (ex 21) [34]
Behavioral disorder (*n* = 1)	Mild intellectual deficiency (*n* = 1)	0.15	0.80	–
Alcoholism (*n* = 1)	–	0.15	0.78	–
Anorexia nervosa (*n* = 1)	–	0.13	0.61	–
Depressive disorder (*n* = 1)	–	0.17	0.87	–

*ASD* Autism spectrum disorders

^a^Cp (g/L), Normal values (NV) 0.18–0.32, borderline values (BV) 0.10–0.17, critical values (CV) ≤0.10

^b^Copper (mg/L), NV 0.88–1.2, BV 0.50–0.87, CV ≤ 0.50

The four cases were examined with careful attention. The urinary Cu/Cr ratios were not increased except for one patient with a ratio value of 14.6 µg/g, slightly above the 3rd quartile of the reference population (12.1 µg/g). Neurological examination did not reveal the usual pattern of WD (no dystonia, dysarthria, or other impairments). No Kayser–Fleischer rings were observed. Brain MRI was normal in three cases. The fourth case was a 35-year-old woman with a particular history of depression with suicidal attempt and cardiac arrest. Subsequently, she developed a syndrome of pure psychic akinesia with the usual Globus pallidus necrotic lesions shown at MRI [23, 24]. These patients receive a regular neurological and psychiatric assessment for four years. They did not exhibit other symptoms of WD.

### Serum ceruloplasmin and copper thresholds for WD screening in hospitalized patients with psychiatric disorders

Using Cp and Cu below the normal values to identify possible WD clearly generates high levels of false positive results (assuming that no patients had WD, the false positive rate was 19%).

## Discussion

Establishing a diagnosis of WD is crucial since early detection and treatment may prevent disease progression and even reverse damage in some patients. Although the lifetime prevalence of psychiatric symptoms in WD patients is unclear, the estimated range is from 30 to 100% of symptomatic patients [25].

This study evaluated the feasibility and relevance of serum Cp and Cu level measures in a small sample size of hospitalized patients with psychiatric disorders (*n* = 269). Conventional WD testing is based on serum ceruloplasmin levels, but a normal ceruloplasmin level cannot exclude Wilson’s disease [10, 26]. The serum copper, usually low, can be elevated as a result of release of free copper from a damaged liver.

Our main findings are the following: (1) a ratio of 1/5 psychiatric patients with decreased Cp and Cu concentrations; and (2) the detection of several heterozygous *ATP7B* mutations in patients with levels of ceruloplasmin and/or serum copper below the normal limits.

Given the small population of patients (*n* = 269), this study had too insufficient statistical power to draw any conclusions about the prevalence of WD in hospitalized patients with psychiatric disorders.

Our results are discussed below:

### Ceruloplasmin and serum copper values

As shown in Table 1, psychiatric diagnosis had no significant effect on Cp and serum Cu levels since only alcoholic patients showed increased levels. This may be easily explained by the consequences of alcoholism on hepatic functions as previously described [27].

More interestingly, values below the normal ranges for ceruloplasmin and serum copper have been noted in 19% of the psychiatric patients tested for WD. Thus, these criteria alone are not differentiating enough to establish a specific diagnosis of WD in patients with psychiatric disorders. Specific management is required to help psychiatrists with the early detection of WD in patients presenting isolated psychiatric symptoms without neurological and/or hepatic impairment. In such cases, exclusion of WD requires normal physical examination, brain MRI, and standard hepatic profile test.

A review of the literature on the psychiatric manifestations of WD reported that Cp was <0.10 g/L in most cases, except for a 15-year-old girl suffering from atypical depression and personality disorders. Her Cp was 0.24 g/L with the increased hepatic enzymes, which led to Kaiser–Fleisher ring research and 24-h urine copper assay to confirm the diagnosis [28]. In a 38-year-old man with severe depression, Cp was 0.12 g/L [29]. Decreased serum Cu (<0.60 mg/L) and increased urinary copper levels were found in all patients (>100 µg/24 h).

Copper homeostasis in the CNS is securely synchronized, and perturbations in brain copper levels are known to underlie the mechanism of a wide spectrum of common neurodegenerative disorders [30]. Hence, it could be very promising to develop this approach in mental health.

## Ceruloplasmin concentrations and pitfalls of interpretation

The substantial intra-individual biological variability (CVi) of serum Cp (CVi = 6.2%) may limit the clinical use of these data [31]. Thus, the accurate assessment of individual Cp concentrations in clinical practice requires two serum Cp measurements.

The standardization of Cp measurements remains an issue despite the availability of common reference material (ERM-DA470) [21, 32]. For instance, Infusino et al. [33] compared two methods for Cp measurement (Roche turbidimetric immunoassay vs. Beckman nephelometric immunoassay). Although a fairly good correlation was observed (*r* = 0.955), a highly significant proportional and constant bias was found: [Turbidimetric method] = 0.572 [Nephelometric method] + 0.05 g/L. Consequently, clinicians should consider thresholds depending on the method used rather than adopting universal standards. Like other groups, we follow this rule in our laboratory.

### Molecular analysis of the ATP7B gene

The study also showed that 4 of the 29 patients who gave their consent and were subject to molecular study (Cp < 0.18 g/L) presented with heterozygous *ATP7B* gene mutation. So far, such heterozygous carriage had been described as asymptomatic. Among these variants, two missense mutations were identified in the 3′ coding region (exon 21): p.Pro1379Ser and p.Thr1434Met, corresponding to the C-terminus of ATP7B which is necessary for protein stability. Usually, the exon 21 is rarely mutated. Both variants were already reported by Cox [22] and Loudianos [34], respectively. They do not seem to affect copper transport function in the hepatocyte [35]. Further studies are needed to confirm these original data.

In previous articles, molecular data revealed over 520 disease-causing mutations and 136 polymorphisms in the *ATP7B* gene along the whole length of the entire coding region (21 exons), as well as in promoter and intronic regions [22, 36–38]. These mutations may cause reduced copper incorporation into Cp and biliary excretion, leading to the accumulation of copper in the liver, brain, and cornea. In France, the mutations are mainly distributed on exons 2, 3, 5, 6, 7, 8, 10, 12, 13, 14, 15, 16, 17, 18, 19, and 20, with a higher level of mutation observed on exons 14, 8, and 13 [22].

## Conclusions

Considering the high rate of false positives in psychiatric population, the diagnosis of WD should be based on a combination of clinical and biological features. According to our study, specific management (e.g., in a reference center) is required when patients show threshold values, or when psychiatric and/or neurological and/or hepatic impairments are combined with borderline biological thresholds. Considering the literature data and this preliminary report, Cp levels ≤0.13 g/L and serum Cu levels ≤0.60 mg/L seem to be the most accurate values to identify potential WD in psychiatric patients with normal hepatic and neurological profiles. However, this preliminary observation remains to be confirmed with further studies. Indeed, the exploration of copper metabolism in mental diseases should be of interest.

## Abbreviations

ATP7B: ATPase, Cu++ transporting, beta polypeptide
CNS: central nervous system
Cp: ceruloplasmin
CV: coefficient of variation
ICD: international classification of diseases
Serum Cu: serum copper
WD: Wilson’s disease
